# C1, a highly potent novel curcumin derivative, binds to tubulin, disrupts microtubule network and induces apoptosis

**DOI:** 10.1042/BSR20160039

**Published:** 2016-04-27

**Authors:** Shalini Srivastava, Satyendra Mishra, Avadhesha Surolia, Dulal Panda

**Affiliations:** * Department of Biosciences & Bioengineering, Indian Institute of Technology Bombay, Mumbai 400076, India; † University and Institute of Advanced Research, Koba Institutional Area, Koba, Gandhinagar 382007, India; ‡ Molecular Biophysics Unit, Indian Institute of Science, Bangalore 560012, India

**Keywords:** anticancer, apoptosis, curcumin, microtubule

## Abstract

We have synthesized a curcumin derivative, 4-{5-(4-hydroxy-3-methoxy-phenyl)-2-[3-(4-hydroxy-3-methoxy-phenyl)-acryloyl]-3-oxo-penta-1,4-dienyl}-piperidine-1-carboxylic acid *tert*-butyl ester (C1) that displays much stronger antiproliferative activity against various types of cancer cells including multidrug resistance cells than curcumin. C1 depolymerized both interphase and mitotic microtubules in MCF-7 cells and also inhibited the reassembly of microtubules in these cells. C1 inhibited the polymerization of purified tubulin, disrupted the lattice structure of microtubules and suppressed their GTPase activity *in vitro*. The compound bound to tubulin with a dissociation constant of 2.8±1 μM and perturbed the secondary structures of tubulin. Further, C1 treatment reduced the expression of Bcl2, increased the expression of Bax and down regulated the level of a key regulator of p53, murine double minute 2 (Mdm2) (S166), in MCF-7 cells. C1 appeared to induce p53 mediated apoptosis in MCF-7 cells. Interestingly, C1 showed more stability in aqueous buffer than curcumin. The results together showed that C1 perturbed microtubule network and inhibited cancer cells proliferation more efficiently than curcumin. The strong antiproliferative activity and improved stability of C1 indicated that the compound may have a potential as an anticancer agent.

## Abstract (precis)

C1 is one of the most potent curcumin analogues identified till date which inhibits proliferation of various cancer cell lines. C1 binds to tubulin and depolymerized microtubules of MCF-7 cells. C1 altered the expression of apoptotic proteins and induces p53-dependent apoptosis.

## INTRODUCTION

Curcumin is known to display antiproliferative activities against various types of cancer cells [1–9]. Curcumin is under phase II clinical trials for the treatment of pancreatic cancer (ClinicalTrials.gov Identifier: NCT00094445), breast cancer (ClinicalTrials.gov Identifier: NCT01740323), colorectal cancer (ClinicalTrials.gov Identifier: NCT00118989) and rectal cancer (ClinicalTrials.gov Identifier: NCT00745134). In addition, curcumin has entered into phase III clinical trials in combination with gemcitabine and celebrex for pancreatic cancer (ClinicalTrials.gov Identifier: NCT00486460). Curcumin was found to be effective with low side effects in clinical trials indicating a potential chemotherapeutic and chemopreventive use of the compound [10–13].

Curcumin perturbs a large number of cellular processes [14]. It targets regulatory molecules including transcription factors, critical signalling molecules, enzymes, growth factors, apoptotic proteins and tubulin [14–19]. Curcumin is reported to exert its activity by blocking the cell at a defined cell cycle stage, inducing autophagic death of the cell, inhibiting the invasion capability of tumour, preventing angiogenesis and by inducing programmed cell death of the cancer cells [15]. Previous studies suggested that tubulin is one of the primary targets for curcumin [16–18]. Curcumin binds to tubulin and inhibits its assembly *in vitro* [16,17]. Curcumin perturbed the dynamic instability of microtubules in MCF-7 cells and induced apoptosis in these cells [18].

The poor absorption, low bioavailability and poor stability of curcumin limit its wider application for treating cancer. Therefore, several attempts were made to use curcumin in combination with adjuvant or to load with nanoparticles for improved anticancer activity [19]. An alternative strategy involves the synthesis of curcumin-derived molecules with a similar backbone but with modified functional groups [13]. Curcumin is a polyphenol, which consists of two α–β unsaturated carbonyl groups with a diketo moiety in the middle. The two hydroxy groups at the two phenyl rings are reported for the activity and the instability of curcumin in aqueous solution [20]. A large number of curcumin analogues have been synthesized and screened for their anticancer activity [21–24].

In this work, curcumin derived compounds modified at the active methylene (C1–C4) have been evaluated. C4 was synthesized earlier (reported as Compound 7) and found to be more potent than curcumin against HeLa cells [17]. In the present study, C1 and C3 were found to display more potent antiproliferative activity than curcumin against MCF-7 cells. Both C1 and C3 inhibited microtubule assembly *in vitro* and disrupted the microtubule network in cells. However, C1 inhibited the proliferation of MCF-7 cells at a lower concentration than C3. Therefore, we sought to elucidate the mechanism of action of C1. C1 bound to tubulin, inhibited and suppressed the GTPase activity of microtubules. In addition, C1 was found to disrupt the secondary structure of tubulin. We provide data suggesting that C1 treatment induced p53 dependent apoptotic pathway in MCF-7 cells. C1 is one of the most potent curcumin derivatives reported so far and the results suggest that C1 might have a potential as an anticancer agent.

## EXPERIMENTAL

### Materials

Sulforhodamine B (SRB), mouse monoclonal anti-α-tubulin IgG, mouse monoclonal anti-β-actin IgG, alkaline phosphatase conjugated anti-mouse IgG, rabbit monoclonal anti-Bax IgG, alkaline phosphatase conjugated anti-rabbit IgG, Hoechst 33258 dyes were purchased from Sigma. Annexin V FITC and propidium iodide apoptosis detection kit was purchased from BD Pharmigen. Alexa flour 568 anti-mouse IgG and FBS were purchased from Molecular probes, Invitrogen. Mouse monoclonal anti-p53 IgG, rabbit polyclonal anti-Bcl2 IgG, rabbit polyclonal anti-PARP (poly ADP ribose polymerase) IgG and mouse monoclonal anti-p21 IgG were purchased from Santa Cruz Biotechnology. Rabbit polyclonal anti-murine double minute 2 (Mdm2; S166) was purchased from Abcam. ^1^H NMR was recorded on a Buker 300 Hz instrument and mass on Applied Biosystem 4700**.** Other reagents used in the study were of analytical grade and were obtained from Sigma or HiMedia.

### Cell culture

Human breast adenocarcinoma (MCF-7), human cervical carcinoma (HeLa), highly metastatic breast adenocarcinoma (MDA-MB-231) and human colorectal carcinoma (HCT 116) cells were procured from National Centre for Cell Science. The multidrug resistant mouse mammary tumour (EMT6/AR1) cells were purchased from Sigma. MCF-7 and HeLa cells were cultured in Eagle's minimal essential medium (MEM) (HiMedia) supplemented with 10% (v/v) FBS and 1% (v/v) antibiotic–antimycotic solution as described earlier [25]. MDA-MB-231 cells were grown in Leibovitz's L-15 medium [26]. EMT6/AR1 cells were grown in MEM medium containing 1 mg/ml doxorubicin [27]. All the cells were cultured at 37°C incubator in humidified chamber of 5% CO_2_.

### Determination of IC_50_ of curcumin analogues in the MCF-7 cells

Curcumin derivatives (C1, C2, C3 and C4) were dissolved in DMSO. MCF-7 cells (1×10^5^ cells/ml) were seeded in a 96 well cell culture plate for 24 h. The medium was then replaced with a fresh medium containing either the vehicle (0.1% DMSO) or different concentrations of C1, C2, C3, C4 and curcumin. The cells were allowed to grow for 48 h, fixed with 50% (tricarboxylic acid) TCA for 1 h at 4°C, then washed and dried completely. Sulforhodamine B (0.4%) was added to the well for 1 h and further washed with 1% acetic acid [28]. After the plate was dried, Tris chloride (10 mM, pH 8.0) was added for 30 min and the reading was taken at 520 nm. The concentration of a compound required to inhibit the proliferation of cells by 50% was defined to be its IC_50_ value. The experiment was performed three times for each curcumin analogue. The IC_50_ values for HeLa, MDA-MB-231, EMT6/AR1 and HCT 116 (p53^++^/p53^−−^) cells were determined similarly after incubating the cells with C1 for one cell cycle. The IC_50_ value of curcumin in EMT6/AR1 was determined as mentioned above.

### Microtubule polymerization assay

Tubulin was purified from goat brain using the protocol as described earlier [29] and the protein concentration was determined by Bradford method [30]. Tubulin (10 μM) was incubated without and with different concentrations (0.1, 0.2, 0.5, 1, 2, 5, 10 and 20 μM) of C1 in PEM buffer [50 mM piperazine-*N*,*N*′-bis(ethanesulfonic acid) (PIPES), pH 6.8, 3 mM MgCl_2_, 1 mM EGTA] with 1 M glutamate at 4°C for 10 min [31]. Subsequently, 1 mM guanosine-5′-triphosphate (GTP) was added and the reaction tube was transferred to SoftMax Spectra Multi Plate reader set at temperature 37°C. The polymerization was monitored for 20 min by measuring the absorbance at 350 nm. C1 (0.1, 0.2, 0.5, 1, 2, 5, 10 and 20 μM) was also monitored under the similar conditions and subtracted from the respective reaction set. Similarly, the effect of different concentrations of C3 (0, 3, 5, 10, 15 and 20 μM) on tubulin assembly was studied. The experiment was done three times for C1 and C3.

### Electron microscopy

MAP rich tubulin was purified by using 4 M glycerol instead of monosodium glutamate [32]. Tubulin (1.5 mg/ml) was incubated in the absence and presence of C1 (10 μM) in PEM buffer on ice. Afterward, 1 mM GTP was added and transferred to water bath set at 37°C for 20 min. The polymers were then fixed with 0.5% glutaraldehyde and spotted on carbon-formvar coated grids (Electron Microscopy Sciences). The grids were then washed with milliQ water and stained with 1% uranyl acetate [33]. The microtubules were then visualized under an electron microscope (JEM 2100 ultra HRTEM instrument at 200 kV).

### Malachite green assay

Tubulin (10 μM) was incubated without and with different concentrations (1, 2, 3, 5 and 10 μM) of C1 for 10 min on ice in PEM buffer with 1 M monosodium glutamate. Similar concentrations of vinblastine were taken as a positive control. Subsequently, 1 mM GTP was added to the reaction mixtures and the reaction mixtures were incubated at 37°C for 10 min. The reaction was stopped by adding 10% (v/v) of 7 M perchloric acid. The amount of inorganic phosphate released was estimated using the standard malachite green assay [31,34]. The experiment was performed three times.

### Circular dichroism

Tubulin was incubated without and with C1 (2 and 4 μM) for 20 min in 10 mM phosphate buffer pH 7.2 at 25°C. The far-UV (195–260 nm) CD spectra were recorded in JASCO J-1500 CD spectrophotometer using a quartz cuvette of 0.1 cm path length. The experiment was done three times. The data obtained were analysed using CDPro software and secondary structure prediction was done using CONTINLL, CDSSTR and SELCON3 [35].

### Gel filtration

The interaction between C1 and tubulin was monitored using size exclusion chromatography. The void volume of the P4 resin was determined using blue dextran. Tubulin (20 μM) was incubated with C1 (40 μM) for 30 min at 25°C in 50 mM PIPES (pH 6.8). Tubulin, C1 and tubulin–C1 complex were loaded individually on to the column and fractions of 200 μl each was collected. Tubulin was monitored using Bradford reagent by measuring absorbance at 595 nm and C1 was detected by monitoring the absorbance at 360 nm. The experiment was performed twice.

### Determination of the binding affinity of C1 to tubulin

Tubulin (3 μM) was incubated in the absence and presence of different concentrations (0.1, 0.2 0.5, 1, 2, 3, 5, 10, 15, 20 and 25 μM) of C1 for 30 min in 50 mM PIPES (pH 6.8) at 25°C. The fluorescence spectra (310–370 nm) were recorded by exciting the reaction mixtures at 295 nm. A cuvette of path length (0.3 cm) was used for the experiment. The inner filter effect correction was performed using the following formula,

$$F_{\mathrm{corrected}}\;=\;F_{\mathrm{observed}}\;\times \;\mathrm{antilog}\;\frac{\left(A_{\mathrm{excitation}\;}+\;A_{\mathrm{emission}}\right)}{2}$$

where *F*_corrected_ is the corrected florescence, *F*_observed_ is the observed fluorescence, *A*_excitation_ is the absorbance of compound at its excitation wavelength (295 nm) and *A*_emission_ is the absorbance at the emission maxima wavelength (334 nm).

The fluorescence intensity values were fitted in the equation,

$$\Delta F\;=\frac{\Delta F\max\left[\left(\left[P_{0}\right]+\left[L_{0}\right]+K_{d}\right)-\sqrt{{\left(\left[P_{0}\right]+\left[L_{0}\right]+K_{d}\right)}^{2}-4\left[P_{0}\right]\left[L_{0}\right]}\right]}{2\left[P_{0}\right]}$$

where, Δ*F* is the change in fluorescence in the presence of C1, Δ*F*_max_ is the maximum difference in the fluorescence intensity when tubulin is fully bound with C1, *P*_0_ and *L*_0_ are the concentrations of tubulin and C1 respectively [31]. The value of the dissociation constant (*K*_d_) was determined using the Graph Pad Prism 5 software. The experiment was done three times.

In a separate experiment, tubulin (3 μM) was incubated in the absence and presence of different concentrations (1–20 μM) of curcumin for 20 min in 50 mM PIPES (pH 6.8) at 25°C. A dissociation constant for the binding of curcumin to tubulin was determined as described above.

### Competition assay

Tubulin (3 μM) was incubated with curcumin (5 μM) for 20 min at 25°C in PIPES buffer (50 mM, pH 6.8). Then, the reaction mixtures were incubated in the absence and presence of different concentrations (2, 5, 10, 15 and 20 μM) of C1 for an additional 10 min. The fluorescence spectra were recorded by exciting the reaction mixtures at 425 nm [16,17]. The difference in the fluorescence intensity of tubulin–curcumin complex in the presence of C1 was calculated and fitted into the formula,

$$K_{i}=EC_{50}/\left(1+\left[L\right]/K_{d}\right)$$

where *K*_i_ is the half-inhibitory concentration of C1, a concentration of C1 required to displace curcumin by 50%, EC_50_ is the value at which fluorescence was decreased to the half in the presence of C, [*L*] is the concentration of C1 and *K*_d_ is the dissociation constant of binding of curcumin to tubulin [36].

### Stability

C1 and curcumin (20 μM) were incubated in PBS for 4 h and the absorbance of the compounds was monitored at 360 and 425 nm, respectively using a SoftMax Spectra Multi Plate reader. The experiment was repeated three times.

### Immunofluorescence microscopy

Immunofluorescence staining was performed as described earlier [31,32,37,38]. Briefly, MCF-7 cells (1×10^5^ cells/ml) were seeded on to coverslips in a 24 well cell culture plates. The media was replaced after 24 h with a new media either containing the vehicle (0.1% DMSO) or C1 (3 and 6 μM). The cells were grown for either 30 min or 24 h at 37°C. The cells were fixed with 3.7% formaldehyde at above mentioned time points. Microtubules were stained using monoclonal anti-α-tubulin mouse IgG and anti-mouse Alexa 568. The staining for p53 and p21 was performed using monoclonal anti-p53 and anti-p21 mouse IgG. DNA was stained using Hoechst 33258 dye. The fluorescence images were captured using TE Eclipse 2000U fluorescence microscope (Nikon) and analysed with Image-Pro Plus software.

### C1 inhibited the reassembly of depolymerized microtubules

MCF-7 cells were grown in a 24-well cell culture plates for 24 h. The media was then replaced with a chilled media and incubated on ice for 30 min. The cold media was replaced with a warmed media containing either the vehicle (0.1% DMSO) or C1 (6 μM) and the cells were incubated at 37°C for 15 and 30 min in a CO_2_ incubator [26,38]. The cells were fixed with formaldehyde at different time intervals and microtubules were immunostained.

### C1 induced apoptosis in MCF-7 cells

MCF-7 cells treated with either a vehicle (0.1% DMSO) or different concentrations (3 and 6 μM) of C1 or curcumin (6 μM) for 48 h. The cells were then stained with Annexin V and propidium iodide [25,31] (BD Biosciences). The data were compensated with the control cells.

### Western blot analysis

MCF-7 cells treated without and with C1 (3 and 6 μM) for 24 h and the cells were lysed [37,38]. The protein concentration was determined by Bradford method using BSA as standard [30]. An equal concentration of protein was loaded on SDS/PAGE and the separated protein was electro-blotted on to PVDF membrane. Immunoblotting was performed using the anti-PARP, anti-p53, anti-p21, anti-Bax, anti-Bcl2 and anti-Mdm2 (S166) IgG. ImageJ software was used for measuring the band intensities in each blot.

## RESULTS

### Chemistry

Knoevenagel condensates of curcumin were prepared as illustrated in Supplementary Scheme S1 as reported previously [39,40]. Knoevenagel condensates of curcumin were synthesize by reaction of curcumin with respective aldehyde (4-formyl-piperidine-1-carboxylic acid *tert*-butyl ester, 4-hydroxy-3-methoxybenzaldehyde; benzaldehyde) in the presence of catalytic amount of piperidine and anhydrous dimethylformamide (DMF) to yield the desired compound C1, C3 and C4. Boc-group from curcumin derivative C1 was deprotected in the presence of trifluoroacetic acid (TFA) and anhydrous CH_2_Cl_2_ to get compound C2 in quantitative yield (Supplementary Scheme S1).

**Figure 1:**
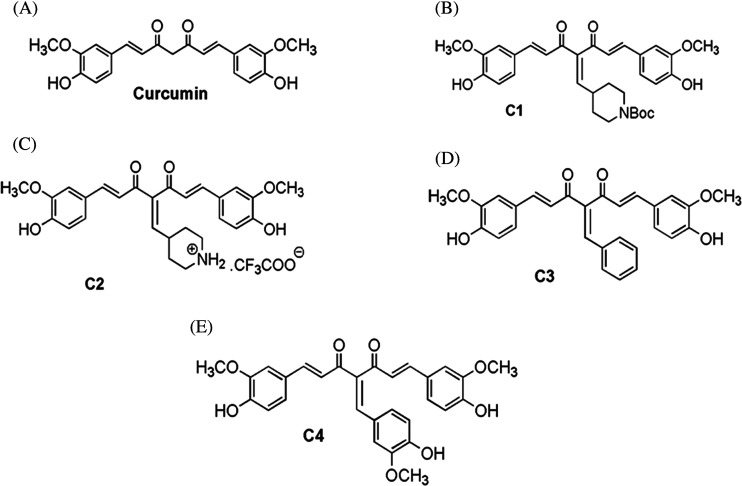
Structure of curcumin and curcumin analogues Curcumin (**A**), 4-{5-(4-hydroxy-3-methoxy-phenyl)-2-[3-(4-hydroxy-3-methoxy-phenyl)-acryloyl]-3-oxo-penta-1,4-dienyl}-piperidine-1-carboxylic acid *tert*-butyl ester, **C1** (**B**), trifluoro-acetate4-{5-(4-hydroxy-3-methoxy-phenyl)-2-[3-(4-hydroxy-3-methoxy-phenyl)-acryloyl]-3-oxo-penta-1,4-dienyl}-piperidinium **C2** (**C**), 4-benzylidene-1,7-bis-(4-hydroxy-3-methoxy-phenyl)-hepta-1,6-diene-3,5-dione **C3** (D) and 4-(4-hydroxy-3-methoxy-benzylidene)-1,7-bis-(4-hydroxy-3-methoxy-phenyl)-hepta-1,6-diene-3,5-dione **C4** (**E**).

The formation of compounds C1, C2, C3 and C4 via Knoevenagel's condensation was validated by the disappearance of aldehydic proton (*δ* 9.80–10.10 ppm) and appearance of singlet proton at 8.0 and 7.85 ppm corresponds to benzylidene (═CH-Ar)─; indicates the structure of desired compounds C3 and C4. In similar fashion appearance of piperidylidene (═CH-piperidine) proton at *δ* 7.91 showed the formation of compound C2. In addition all the Knoevenagel condensates of curcumin were characterized by ESI-MS also (Supplementary Information NMR/MS spectral data Supplementary Figures S1–S8). The purity of compounds is >95% as determined by HPLC. The chemical structures of curcumin and Knoevenagel condensates of curcumin (C1, C2, C3 and C4) are shown in Figure 1.

### C1 and C3 inhibited the proliferation of MCF-7 cells more potently than curcumin

The antiproliferative potential of curcumin analogues (C1–C4) were determined using MCF-7 cells. The half-maximal proliferation inhibitory concentration (IC_50_) of C1, C2, C3 and C4 was found to be 1.5±0.7, 26.2±3, 2.9±0.4 and 6.3±0.2 μM, respectively (Supplementary Figure S9, Table 1) in MCF-7 cells. Consistent with the previous studies [18,41,42], curcumin inhibited the proliferation of MCF-7 cells with an IC_50_ value of 17.1±0.7 μM indicating that C1 and C3 were more potent inhibitors of MCF-7 proliferation than curcumin.

**Table 1:** Half-maximal inhibitory concentration (IC_50_) of curcumin analogues in MCF-7 cells The data represent an average IC_50_ value from three independent sets of experiment with S.D. (±).

Compounds	Half-maximal inhibitory concentration IC_50_ (μM)
C1	1.5±0.7
C2	26.2±3
C3	2.9±0.4
C4	6.3±0.2
Curcumin	17.1±0.7

### C1 inhibited tubulin assembly more strongly than C3 *in vitro*

**Figure 2:**
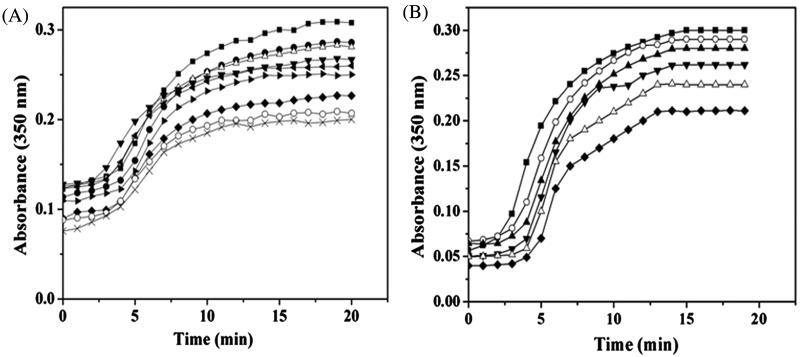
C1 inhibited tubulin assembly more strongly than C3 (**A**) Tubulin was polymerized in the absence (■) and presence of different concentrations of **C1** [0.1 μM (●), 0.2 μM (Δ), 0.5 μM (▼), 1 μM (◀), 2 μM (**▶**), 5 μM (◆), 10 μM (○) and 20 μM (×)] in PEM buffer with 1 M monosodium glutamate and 1 mM GTP for 20 min at 37°C. (**B**) Tubulin polymerization was done under similar conditions as mentioned above in the absence (■) and presence of **C3** 3 μM (○), 5 μM (▲), 10 μM (▼), 15 μM (Δ) and 20 μM (◆). The experiment was done three times for **C1** and **C3**. Shown is one of the independent sets of experiments.

Curcumin was found to inhibit tubulin assembly *in vitro* [16,17], therefore, we checked the effect of C1 and C3 on the assembly of tubulin *in vitro*. Both C1 (Figure 2A) and C3 (Figure 2B) inhibited the glutamate-induced assembly of purified tubulin. The polymerization of tubulin was inhibited by 37±2, 43±1.5 and 53±3% in the presence of 5, 10 and 20 μM C1 respectively. However, 5, 10 and 20 μM C3, inhibited the polymerization of tubulin by 18±4, 26±8 and 45±4%, respectively, indicating that C1 inhibited tubulin polymerization more strongly than C3. Both C1 and C3 (6 μM) depolymerized microtubules in MCF-7 cells whereas the vehicle treated cells showed typical microtubule network (Supplementary Figure S10). The results indicated that like curcumin, C1 and C3 also depolymerized microtubules in cells. Since C1 and C3 both inhibited microtubule assembly *in vitro* and in cells; we sought to elucidate the mechanism of action of C1 because it is more potent than C3.

### C1 inhibited tubulin assembly and GTPase activity of microtubules

**Figure 3:**
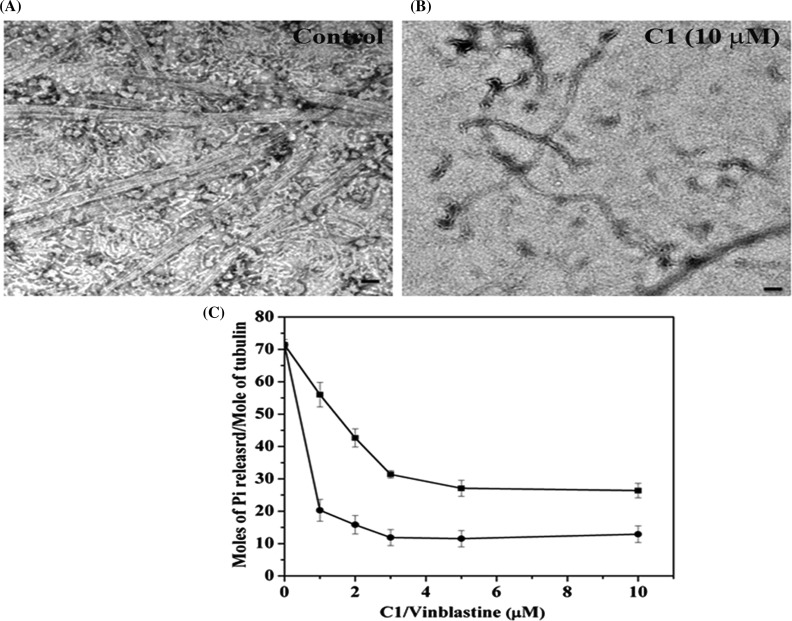
C1 inhibited the assembly and GTPase activity of microtubules Electron micrograph of MAP rich microtubules polymerized with 1 mM GTP in the absence (**A**) and presence of **C1** (10 μM, **B**). The scale bar indicates 50 nm. (**C**) **C1** inhibited the GTPase activity of microtubules. Tubulin was polymerized in the presence of increasing concentrations (1, 2, 3, 5 and 10 μM) of **C1** (■) and vinblastine (●) in PEM buffer for 10 min at 37°C with 1 M monosodium glutamate and 1 mM GTP. The data represent an average of three sets; error bars are S.D.

MAP-rich tubulin was polymerized to from microtubules (Figure 3A). In the absence of C1, microtubules were found in most of the grid areas whereas, in the presence of 10 μM C1, few microtubules were observed per field of view (Figures 3A and 3B). Control microtubules were straight and intact flattened tubes [33]. In contrast, the microtubules formed in the presence of C1, were curved and no distinct protofilaments could be seen (Figure 3B). Similarly, when pure tubulin was polymerized, fewer microtubules were observed per microscopic field in the presence of C1 than its absence. The results indicated that C1 inhibited the assembly of microtubules *in vitro*.

Microtubules show GTPase activity, which is critical for its assembly and dynamics [43]. C1 and vinblastine (a known inhibitor of microtubules) inhibited the GTPase activity of microtubules in a concentration dependent manner (Figure 3C). For example, 10 μM C1 suppressed the GTPase activity by 64±5.2% and vinblastine (10 μM) reduced the GTPase activity by 84±3.5%.

### C1 disrupted the secondary structure of tubulin

**Figure 4:**
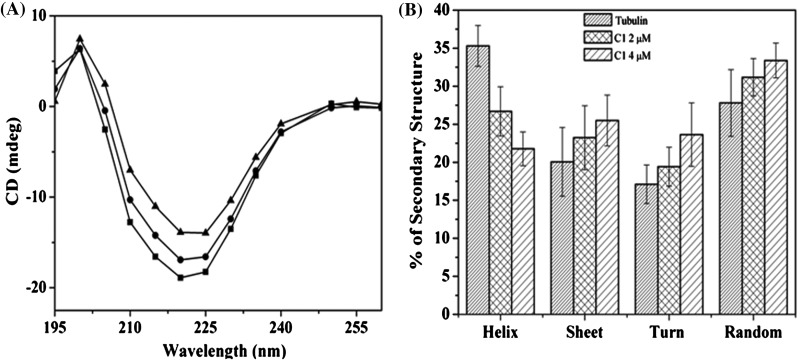
C1 perturbed the secondary structure of tubulin (**A**) Tubulin was incubated without (■) and with **C1**, 2 μM (●) and 4 μM (▲) and the far UV (195–260 nm) signal was recorded using CD spectrophotometer. The experiment was done three times, shown is one of the independent sets. (**B**) The secondary structure (α-helix, sheet, turn and random coil) content of tubulin in the absence and presence of **C1** was determined using CDPro software and plotted as mean with ±S.D.

An analysis of the secondary structure of tubulin by circular dichroism spectroscopy suggested that tubulin contains 35±2.7% helix, 21±4.5% sheet and 45±5.5% turns and random coils as described previously [44] (Figure 4A). C1 (2 and 4 μM) significantly decreased the α-helix content of tubulin. For example, the α-helix content decreased from 35±2.7 to 27±3.2 and 22±2.2 in the presence of 2 and 4 μM C1 respectively. The turn and random coil contents of tubulin were found to increase in the presence of 4 μM C1 indicating that the binding of C1 alters the secondary structure of tubulin (Figure 4B).

### C1 binds to tubulin *in vitro*

**Figure 5:**
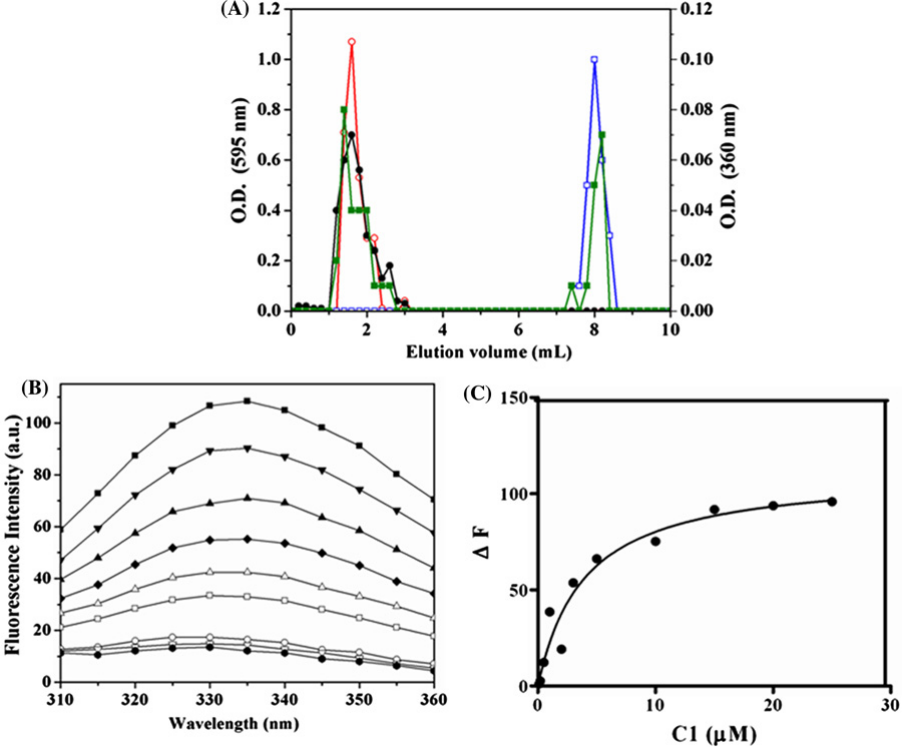
C1 binds to purified tubulin *in vitro* (**A**) The interaction between **C1** and tubulin was monitored using size exclusion chromatography. Tubulin (20 μM) was incubated with **C1** (40 μM). The elution profiles of tubulin (○), free **C1** (□), the mixture of **C1** and tubulin are shown. In the mixture of **C1** and tubulin, the elution of **C1** (■) and tubulin (●) are shown. Tubulin was monitored using Bradford reagent and **C1** was monitored by absorbance at 360 nm respectively. The experiment was performed twice. (**B**) **C1** inhibited the tryptophan fluorescence of tubulin. Tubulin was incubated without (■) and with different concentrations of **C1** [1 μM (▼), 2 μM (▲), 3 μM (◆) 5 μM (Δ) 10 μM (□) 15 μM (○) 20 μM (∇) and 25 μM (●)] for 30 min at 25°C. Fluorescence spectra were recorded by exciting the reaction mixture at 295 nm. (**C**) **C1** quenched the tryptophan fluorescence of tubulin in a concentration dependent manner.

The binding of C1 to tubulin was monitored using size exclusion chromatography. Tubulin and C1 were found to elute at 2 and 8.2 ml respectively (Figure 5A). When a mixture of tubulin and C1 was loaded on to the same column; C1 eluted in two different fractions namely at 2 and 8.2 ml. C1 co-eluted with tubulin suggesting that C1 interacts with tubulin *in vitro.* The affinity of the interaction between C1 and tubulin was determined by monitoring the quenching of the intrinsic tryptophan fluorescence of tubulin. C1 reduced the tryptophan fluorescence of tubulin (Figure 5B) and a dissociation constant for the binding of C1 to tubulin was estimated to be 2.8 ±1 μM (*R*^2^=0.99) (Figure 5C). Curcumin also decreased the tryptophan fluorescence of tubulin and a dissociation constant for the interaction was determined to be 5.2±0.8 μM (*R*^2^=0.99) (Supplementary Figures S11A and S11B).

### C1 displaced the bound curcumin from tubulin

**Figure 6:**
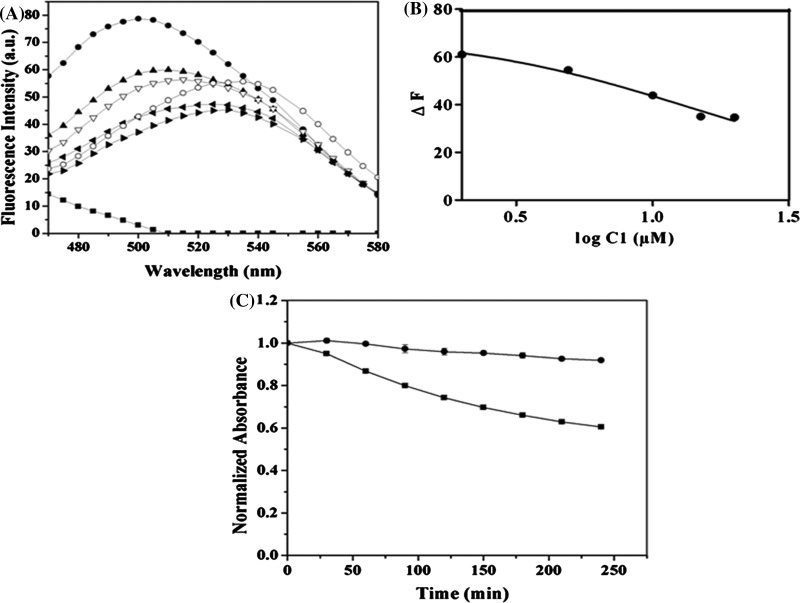
C1 displaced the bound curcumin from the tubulin–curcumin complex (**A**) **C1** decreased the fluorescence of tubulin–curcumin complex. Tubulin was incubated in the absence (■) and presence of curcumin (5 μM) (●) for 20 min. The reaction mixtures were then incubated without or with different concentrations of **C1** [2 μM (▲), 5 μM (∇), 10 μM (◀), 15 μM (▶) and 20 μM (○)] for an additional 10 min. The emission spectra were recorded using 425 nm as the excitation wavelength. (**B**) The difference in fluorescence intensity (Δ*F*) was plotted against the logarithmic value of **C1** concentrations. The experiment was performed three times. (**C**) The absorbance of **C1** (●) and curcumin (■) was taken at 360 and 425 nm respectively for 4 h and was normalized to 1. The experiment was done three times, shown the mean with ±S.D.

Curcumin fluoresces strongly upon binding to tubulin [16,17]. Therefore, we checked whether C1 could inhibit the fluorescence of curcumin–tubulin complex. C1 decreased the fluorescence intensity of curcumin–tubulin complex in a concentration dependent manner (Figure 6A). In addition, there was a red shift in the spectra. The result showed that C1 could displace the bound curcumin from tubulin–curcumin complex. Assuming one binding site for C1 on tubulin, a *K*_i_ value was estimated to be 8±2 μM (*R*^2^=0.99) (Figure 6B).

### C1 is more stable than curcumin in PBS

Both C1 and curcumin were dissolved in PBS, pH 7.4 and the absorbance was recorded for 4 h at 30 min interval. The results showed that there was a significant decrease in the absorbance of curcumin (Figure 6C). For example, the absorbance of curcumin was found to decrease by 22±4 and 40±5% at 2 and 4 h respectively. In contrast, the absorbance of C1 decreased by 5±0.5 and 8±1% at 2 and 4 h, respectively suggesting that C1 is significantly more stable in physiological buffer than curcumin.

### A short exposure of C1 disrupted microtubule network in MCF-7 cells

**Figure 7:**
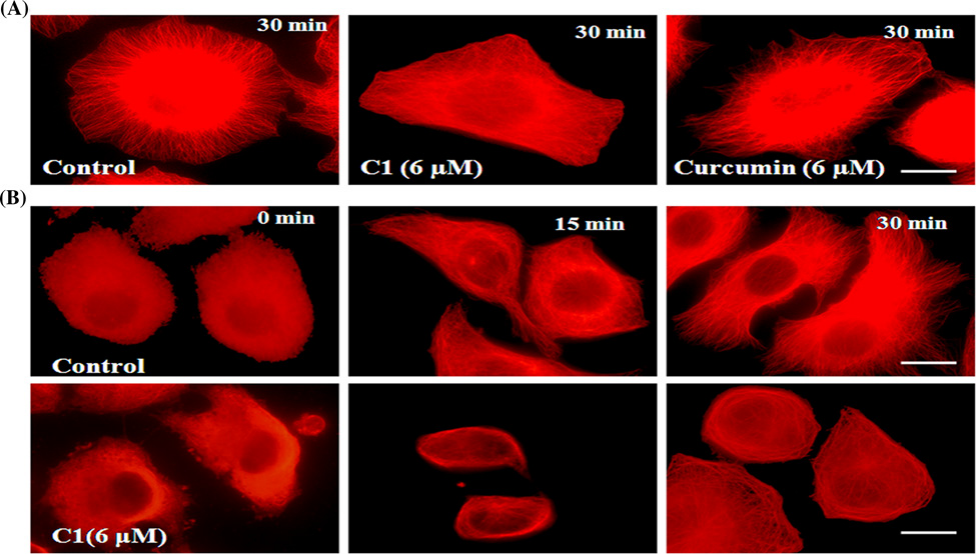
(**A**) A short exposure of C1 to the MCF-7 cells depolymerized microtubules and prevented its reassembly MCF-7 cells were incubated with vehicle (0.1% DMSO) and **C1** (6 μM) for 30 min. Curcumin (6 μM) was taken for comparison. Microtubules were stained with Alexa 568 as a secondary antibody. (**B**) **C1** inhibited the reassembly of depolymerized microtubules. MCF-7 cells seeded on to coverslips were depolymerized by incubation on ice for 30 min. MCF-7 cells were then incubated with warm media containing vehicle (0.1%) and **C1** (6 μM) at 37°C. Cells containing vehicle (0.1%, the upper panel) and **C1** (6 μM, the lower panel) were fixed at 0, 15 and 30 min after incubation and processed for immunostaining using anti-α-tubulin IgG. Scale bar=10 μm.

MCF-7 cells were incubated with C1 (6 μM) for 30 min. C1 depolymerized the microtubules at the cell cortex as evident by the diffused staining of α-tubulin (Figure 7A, C1 6 μM). In the vehicle treated (0.1% DMSO) cells, a typical network of microtubules in interphase cells was observed (Figure 7A, control). A brief (30 min) exposure of 6 μM curcumin did not efficiently depolymerize the microtubules in MCF-7 cells (Figure 7A, curcumin 6 μM) indicating that C1 was more effective in targeting microtubules than curcumin at the similar concentration.

### C1 prevented the reassembly of cold depolymerized microtubules in MCF-7 cells

Upon 30 min incubation on ice, microtubules of the MCF-7 cells got depolymerized. After 15 min incubation at 37°C, a significant reassembly of microtubules occurred in the control cells. Microtubules of the control cells were fully recovered and formed a well-defined network after 30 min of incubation at 37°C (Figure 7B, upper panel). In contrast, the reassembly of microtubules did not occur in the C1 treated cells even after 30 min incubation at 37°C (Figure 7B, lower panel). The result indicated that C1 prevents the reassembly of microtubules in the MCF-7 cells.

### C1 induced apoptosis in MCF-7 cells

**Figure 8:**
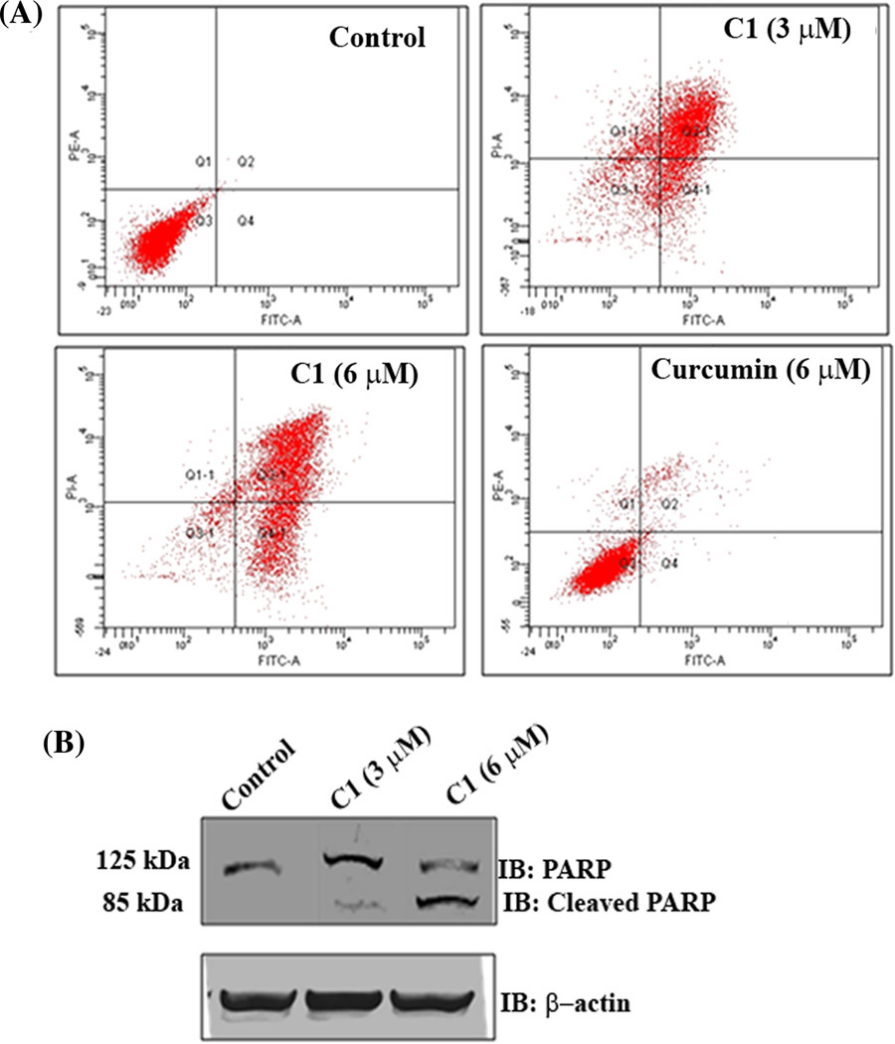
C1 induced apoptosis in MCF-7 cells (**A**) MCF-7 cells were incubated without and with **C1** (3 and 6 μM) or curcumin (6 μM) for 48 h. Then, the cells were stained with Annexin V FITC and propidium iodide and analysed using flow cytometry. The quadrants represent (Q1: Necrotic, Q2: Late apoptosis, Q3: Live, Q4: Early apoptosis). Curcumin (6 μM) was taken for comparison. One of the four experiments is shown. (**B**) **C1** cleaved PARP (125 kDa). MCF-7 cells were incubated without or with **C1** (3 and 6 μM) for 24 h. Shown is one of the blots from three experiments.

C1 treatment induced apoptosis in MCF-7 cells as evident by flow cytometry analysis (Figure 8A). In control cells (0.1% DMSO), the live and dead cells were found to be 95±3 and 4±3% respectively. In the presence of 3 and 6 μM C1, 20±4 and 8±2% of the cells were live and 74±6 and 91±3% of the cells were dead respectively. In the presence of 6 μM curcumin, 80±4.5 and 18±3% cells were live and dead cells respectively (Table 2). A cleavage of PARP protein indicates apoptosis [45]. C1 (6 μM) treatment generated a cleaved fragment of 85 kDa indicating that C1 induced apoptosis in MCF-7 cells (Figure 8B).

**Table 2:** The percentage of live and dead cells was determined from flow cytometry Data are average of four experiments and ± indicates S.D.

	Live (%)	Dead (%)
Control cells	95±3	4±3
C1 (3 μM)	20±4	74±6
C1 (6 μM)	8±2	91±3
Curcumin (6 μM)	80±4.5	18±3

### C1 induced a p53 dependent apoptotic pathway

**Figure 9:**
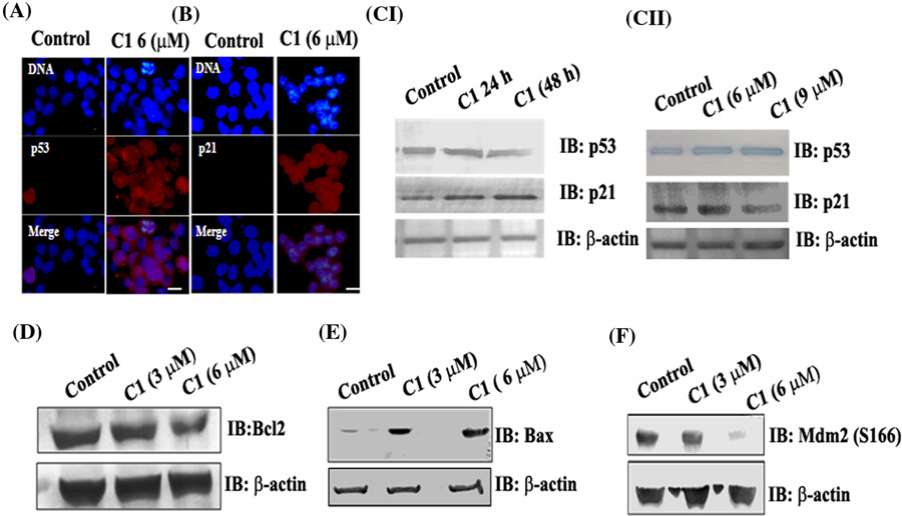
C1 induced p53 dependent apoptosis pathway **C1** increased the localization of p53 (**A**) and p21 (**B**) into the nucleus. MCF-7 cells were incubated with vehicle and **C1** (6 μM) for 24 h. The scale bar is 10 μm. (**C**) Effects of C1 on the expression level of p53 and p21 in MCF-7 cells. MCF-7 cells were treated with 6 μM C1 for 24 and 48 (I). In another experiment, MCF-7 cells were treated with 6 and 9 μM C1 for 24 (II). The expression level of p53 and p21 were quantified by Western blotting. The level of Bcl2 (**D**), Bax (**E**) and Mdm2 (S166) (**F**) were also estimated by Western blotting. Each experiment was performed three times, shown is one of them.

Curcumin is reported to induce apoptosis by activating p53 [46,47]. The activation of p53 is related to its translocation into the nucleus. In C1 treated cells, p53 staining was found in the nuclei of 85±6% of the cells whereas only 12±5% of the vehicle treated cells showed nuclear staining of p53 (Figure 9A, Supplementary Figure S12A). p21 is a downstream protein of p53 activated pathway. The nuclear localization of p21 strongly increased in the C1 treated cells (78±8%) as compared with the vehicle treated cells (10±5%) (Figure 9B, Supplementary Figure S12A). We next checked whether the increased localization to the nucleus was due to an increased expression level of p53 and p21. The effects of C1 on the expression level of p53 and p21 at different times and concentrations were measured by Western blotting (9CI and CII). C1 treatment increased the expression level of p53 and p21 (9 CI and CII). For example, the level of p53 increased by 21±5 (*P*=0.001) and 29±4% (*P*=0.0005) and the level of p21 increased by 27±4 (*P*=0.002) and 36±3% (*P*=0.0004) at 24 and 48 h, respectively, in 6 μM C1 treated cells as compared with the control (9CI). However, the level of p53 increased by 20±4% (*P*=0.001) and 27±3% (*P*=0.0001) when cells were treated with 6 and 9 μM C1, respectively for 24 h. The level of p21 was increased by 19±5% (*P*=0.003) and 26±3% (*P*=0.00012) (9CII). The activation of p53 is reported to regulate apoptosis in several ways [47]. The p53 activation can alter the expression level of protein Bcl-x_L_ and Bax [48,49]. Therefore, the expression level of Bax and Bcl-2 in C1 treated cells were examined. The expression level of anti-apoptotic protein Bcl-2 decreased by 85±7% in 6 μM C1 treated cells than the vehicle treated cells (Figure 9D). Interestingly, the level of the pro-apoptotic protein Bax was increased by 48±15 and 125±20% in the 3 and 6 μM of C1 treated cells as compared with the control cells (Figure 9E, Supplementary Figure S12B). The result suggested that p53 activation in C1 treated cells modulated the expression level of Bcl-2 and Bax. Mdm2 is a primary inhibitor of p53 [50]. The phosphorylation of Mdm2 on Ser^166^ and Ser^186^ positions has been reported to diminish the cellular level as well as the transcriptional activity of p53 [51,52]. The level of Mdm2 (S166) decreased by 14±2 and 45±5% in the presence of 3 and 6 μM C1, respectively with respect to the vehicle treated cells (Figure 9F, Supplementary Figure S12C). The result suggested that C1 treatment reduced the inhibitory activity of Mdm2 and increased the activity of p53 in MCF-7 cells.

### C1 inhibited the proliferation of various cancer cell lines

**Figure 10:**
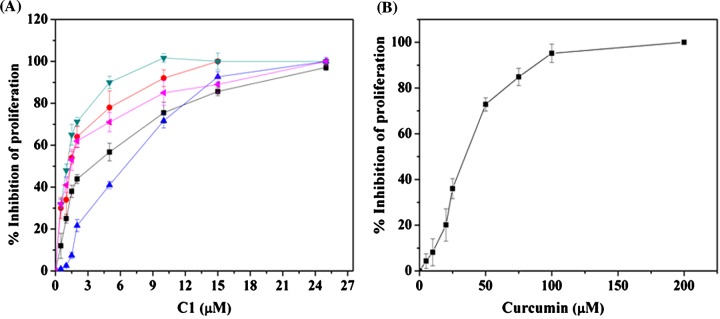
C1 inhibited the proliferation of different types of cancer cells (**A**) C1 inhibited the proliferation of various cancer cells. HeLa (■), MDA-MB-231 (●), EMT6/AR1 (▲), HCT116 p53^++^ (▼) and HCT116 p53^−−^(◀) cells were incubated with different concentrations of **C1** for one cell cycle and sulforhodamine B assay was performed to calculate the percentage inhibition of proliferation. (**B**) EMT6/AR1 cell were incubated with different concentrations of curcumin and processed for sulforhodamine B assay. Shown is an average of three sets and error bar represents S.D. (±) in both cases.

The antiproliferative potential of C1 against human cervical carcinoma (HeLa), highly metastatic breast adenocarcinoma (MDA-MB-231), human colorectal carcinoma (HCT 116 p53^++^ and HCT 116 p53^−−^) and multidrug resistant mouse mammary tumour (EMT6/AR1) cells was evaluated (Figure 10A). The IC_50_ value was determined to be 2.3±0.8, 2.3±0.5, 1.2±0.3, 2.3±0.7 and 7.2±0.3 μM for HeLa, MDA-MB-231, HCT 116 p53^++^, HCT 116 p53^−−^ and EMT6/AR1 respectively (Table 3). The IC_50_ of curcumin for EMT6/AR1 was determined to be 35±2 μM (Figure 10B). The result suggested that C1 is highly effective in targeting various cancer cell lines.

**Table 3:** Half-inhibitory proliferation concentration (IC_50_) of C1 in HeLa, MDA-MB-231, HCT 116 p53^++^, HCT 116 p53^−−^, EMT6/AR1 and MCF-7 cell lines The data represent average IC_50_ values from three independent experiments with S.D. (±).

Cancer cell lines	IC_50_ (μM)
HeLa	2.3±0.8
MDA-MB-231	2.3±0.5
HCT116 p53^++^	1.2±0.3
HCT116 p53^−−^	2.3±0.7
EMT6/AR1	7.2±0.3
MCF-7	1.5±0.7

## DISCUSSION

C1, a curcumin-derived compound, was found to be ∼10 times more potent than curcumin in inhibiting the proliferation of MCF-7 cells. C1 also potently inhibited various cancer cells such as MDA-MB-231, HCT 116 and HeLa cells with IC_50_ values of 2.3±0.5, 1.2±0.3 and 2.3±0.8 μM respectively. In comparison, curcumin exhibited IC_50_ values of 38–45 μM in MDA-MB-231, 43.3 μM in HCT 116 cells and 18 μM in HeLa cells [17,41,42]. Importantly, C1 showed ∼5 times more efficacy than curcumin in inhibiting the proliferation of a multidrug resistant mouse mammary tumour cells, EMT6/AR1 indicating that C1 is more effective than curcumin in inhibiting the proliferation of various types of cancer cells.

Similar to curcumin [16–18], C1 also bound to purified tubulin. The dissociation constant (*K*_d_) for the binding of C1 to tubulin was found to be 2.8 ±1 μM whereas curcumin bound to tubulin with a *K*_d_ of 5.2±0.8 μM indicating that C1 binds to tubulin with a higher affinity than curcumin. C1 inhibited tubulin polymerization *in vitro*. C1 (20 μM) caused 53% inhibition in polymerization whereas 100 μM curcumin caused 59% inhibition of tubulin polymerization [18]. In addition, C1 significantly reduced the GTPase activity of tubulin. C1 depolymerized microtubules in MCF-7 more efficiently than curcumin and also inhibited the reassembly of microtubules in MCF-7 cells. The FACS analyses showed that C1 treated MCF-7 cells were undergoing apoptosis. Though the apoptotic pathway triggered by C1 was similar as curcumin [18,46,47], the apoptotic effect was significantly higher in C1 treated cells than that of curcumin treated cells. For example, ∼80 and 8% of the MCF-7 cells were live in the presence of 6 μM curcumin and C1 respectively. The data together suggested that C1 displayed a strikingly improved antiproliferative activity as compared with curcumin.

### Structure–activity relationship

Curcumin analogues were synthesized by modifying curcumin at the active methylene position and it has been found to be the most potent among the analogues screened. The absorbance of curcumin and C1 reduced by 40±5 and 8±1% at 4 h in the physiological buffer suggesting that C1 is significantly more stable than curcumin. The presence of N-Boc group may stabilize the structure by blocking an attack either by an electrophile or a nucleophile. C1 displayed lower IC_50_ (1.5±0.7) than C2 (26. 2±2.5) in MCF-7 cells; C1 has N-Boc group instead of an amino group as in the case of C2. The amino group in C2 may be available for a reaction with a nucleophile. Also, the benzene derivative of curcumin, C3 (2.9±0.35) and C4 (6.3±0.20) were more effective in inhibiting the proliferation of MCF-7 cells than curcumin (17.1±0.7). The findings suggested that the Knoevenagel condensates of curcumin are more potent and 10-fold increase in the activity of C1 could be partly due to its increased stability in solution than curcumin.

### Mechanism of action

C1 disrupted the structure of microtubules and did not allow the formation of proper microtubules. C1 significantly decreased the α-helix content and increased the random coil and turns in tubulin. The inhibition of tubulin assembly could be due to the perturbed secondary structure of tubulin in the presence of C1. In MCF-7 cells, a brief exposure (30 min) of C1 caused a significant depolymerization of the interphase microtubules indicating that microtubules are likely to be the primary targets of C1. A perturbation of microtubule assembly dynamics led to various cellular defects such as transport of proteins, unfocused mitotic spindles, mitotic block and cell death [53]. Microtubules are also suggested to be involved in the translocation of tumour suppressor gene p53 into nucleus [54,55] leading to the induction of apoptosis [18,55–57]. The disruption of microtubule network was reported to increase the nuclear translocation and expression of p53 and p21 [48]. C1 treatment modestly increased the level of p53 expression. However, it strongly increased the nuclear translocation of p53. The increased nuclear translocation of p53 induced apoptosis in MCF-7 cells. Further, C1 inhibited the proliferation of HCT 116 p53^−−^ and HCT 116 p53^++^ cells with an IC_50_ value of 2.3±0.7 and 1.2±0.3 μM, respectively supporting the suggestion that p53 is involved in mediating apoptosis in C1 treated MCF-7 cells. Though, an alternative cell death mechanism was activated in HCT 116 p53^−−^ cells upon C1 treatment, which caused the inhibition of the proliferation of HCT 116 p53^−−^ cells. In addition, C1 altered the expression of various p53 dependent apoptotic proteins such as Bax and Bcl2. The master regulator of p53 activity, Mdm2 (S166), was found to be down regulated in the presence of C1. Upon phosphorylation by AKT/PI3K, Mdm2 is known to enter into the nucleus and to regulate the activity of p53 [52,58–60]. It might be possible that C1 in addition to targeting microtubules also targets the AKT/PI3K and induces apoptosis as suggested earlier for curcumin [61,62]. Therefore, it is difficult to rule out the possibility of other cellular targets for C1; however, the results suggested that microtubules are the major targets of C1.

## CONCLUSION

In the present study, we have identified a highly potent curcumin analogue, C1. The compound disrupted microtubules and induced p53 dependent apoptotic cell death in MCF-7 cells. C1 could target various cancer cells at low concentration suggesting that the compound may have a strong anticancer potential.

## Abbreviations

GTP: guanosine-5′-triphosphate
Mdm2: murine double minute 2
MEM: Eagle's minimal essential medium
PARP: poly ADP ribose polymerase
PIPES: piperazine-*N*,*N*′-bis(ethanesulfonic acid)
